# Relationships between task awareness, comprehension strategies, and literacy outcomes

**DOI:** 10.3389/fpsyg.2023.1056457

**Published:** 2023-05-03

**Authors:** Karyn P. Higgs, Alecia M. Santuzzi, Cody Gibson, Ryan D. Kopatich, Daniel P. Feller, Joseph P. Magliano

**Affiliations:** ^1^Department of Psychology, Northern Illinois University, DeKalb, IL, United States; ^2^Augustana College, Rock Island, IL, United States; ^3^Department of Learning Sciences, Georgia State University, Atlanta, GA, United States

**Keywords:** reading comprehension, task awareness, task-oriented reading, comprehension strategies, college reading

## Abstract

Reading is typically guided by a task or goal (e.g., studying for a test, writing a paper). A reader’s task awareness arises from their mental representation of the task and plays an important role in guiding reading processes, ultimately influencing comprehension outcomes and task success. As such, a better understanding of how task awareness arises and how it affects comprehension is needed. The present study tested the Task Awareness Mediation Hypothesis. This hypothesis assumes that the strategies that support reading comprehension (e.g., paraphrasing, bridging, and elaborative strategies) also support a reader’s task awareness while engaged in a literacy task. Further, it assumes that the reader’s level of task awareness partially mediates the relationship between these comprehension strategies and a comprehension outcome. At two different time points in a semester, college students completed an assessment of their propensity to engage in comprehension strategies and a complex academic literacy task that provided a measure of comprehension outcomes and an assessment of task awareness. Indirect effects analyses provided evidence for the Task Awareness Mediation Hypothesis showing that the propensity to engage in paraphrasing and elaboration was positively predictive of task awareness, and that task awareness mediated the relationships between these comprehension strategies and performance on the complex academic literacy task. These results indicate that task awareness has complex relationships with comprehension strategies and performance on academic literacy tasks and warrants further consideration as a possible malleable factor to improve student success.

## Introduction

Reading often occurs in the context of a task that requires one to use texts to solve a problem (Snow and The RAND Reading Study Group, 2002; McCrudden et al., 2010; Britt et al., 2018). These tasks provide the context people use to develop goals and strategies to accomplish the task (McCrudden and Schraw, 2007). As such, there is research directed at understanding how tasks and goals affect processing during reading and reading outcomes (e.g., Wiley and Voss, 1999; van den Broek et al., 2001; Snow and The RAND Reading Study Group, 2002; Kaakinen and Hyönä, 2005; McCrudden and Schraw, 2007; McCrudden et al., 2010; Rouet and Britt, 2011; Vidal-Abarca et al., 2011; Higgs et al., 2017; Britt et al., 2018). There is an increasing body of evidence demonstrating that tasks affect reading comprehension in a variety of ways. For example, task instructions affect comprehension outcomes (Wiley and Voss, 1999; Bråten and Strømsø, 2009), what text content readers attend to and remember (Reynolds, 1992; McCrudden and Schraw, 2007), inference processes (Magliano et al., 1999; Narvaez et al., 1999; van den Broek et al., 2001; Linderholm and van den Broek, 2002), and the strategies and behaviors that readers engage in during reading (Cerdán and Vidal-Abarca, 2008; Cerdán et al., 2009; Higgs et al., 2017).

Interest in the role of tasks during reading has arisen in a variety of domains of research within psychology and educational psychology. Several theoretical frameworks of reading comprehension have been proposed to explain the relationship between tasks, texts, and the reader (Snow and The RAND Reading Study Group, 2002; Rouet, 2006; McCrudden and Schraw, 2007; Britt et al., 2018). Further, tasks play an influential role in theories of self-regulated learning (e.g., Butler and Winne, 1995; Winne and Hadwin, 1998, 2008; Pintrich, 2000; Zimmerman, 2002). A unifying feature of these frameworks is that they assume that readers construct a mental representation of a task, which we refer to as a *task* model (Rouet, 2006). This task model reflects the reader’s understanding of a task and guides reading processes as they comprehend texts (e.g., Britt et al., 2018, 2022). While it is well demonstrated that tasks affect reading processes, less is known about the processes that affect how readers construct a task model. Insights into the processes that affect the task model should in turn establish a better understanding of how they affect processing on a literacy task.

In the present study, we were specifically interested in *task awareness* which arises from the reader’s task model and reflects what is accessed from the task model while the reader engages in a literacy activity. Frameworks of reading and self-regulated learning either implicitly or explicitly assume that task awareness (task knowledge accessed during learning) helps to guide processing during reading (Winne and Hadwin, 1998, 2008; Rouet, 2006; McCrudden and Schraw, 2007; Rouet and Britt, 2011; Britt et al., 2018). When reading for a specific purpose, readers must adapt their strategies to the demands of the task to construct a mental representation of the texts(s) that supports task performance. To regulate their learning, readers must maintain an awareness of these task demands. Given the importance of task awareness in learning from texts, understanding the factors that affect a reader’s construction of a task model and task awareness can provide a means of supporting student success. One possibility is that the same comprehension strategies that support the construction of a mental model of text content during reading also operate in support of constructing and updating a task model from which task awareness arises. Literacy activities generally involve understanding explicit content, engaging in elaborative processes, and integrating the information that is consistent with the literacy task (OECD, 2018). Theories of comprehension assume that comprehension strategies such as, paraphrasing, and generating bridging and elaborative inferences play an important role in the process of constructing a mental representation of a text that supports comprehension (McNamara and Magliano, 2009b). Similarly, building and updating a task model involves interpreting instructions, activating knowledge, assessing the relevance of activated knowledge, and integrating this information (Winne and Hadwin, 2008; Schellings and Broekkamp, 2011; Britt et al., 2018). As such, we argue that these three comprehension processes (paraphrasing and bridging and elaborative inferences) should also support the construction of a task model, and therefore, should be related to task awareness.

### Constructing a task model and task awareness

Awareness of a task and the construction of the task model start with developing an understanding of the larger context in which the task is given. The context includes explicitly stated task instructions, information about the requestor or audience, information related to the self (e.g., knowledge, skills, competence assessments), and available supports and constraints (Winne and Hadwin, 1998, 2008; Britt et al., 2018). Based on this initial representation, the reader then constructs a personalized task model that includes the reader’s understanding of what the task outcome(s) should look like (goal states), subgoals, and plans and strategies for obtaining them (Winne and Hadwin, 1998, 2008; Britt et al., 2018, 2022). Awareness of the task during reading arises from readers accessing this task model and guides decisions and actions throughout reading (e.g., selective attention, processing decisions, strategy deployment) as readers construct their mental representation of a text (i.e., a situation model).

Readers also utilize their understanding and awareness of the task to monitor and evaluate progress toward their represented task outcome. As a result of these evaluations, they may engage in additional actions (e.g., additional effort, strategy changes) to achieve their goal. Importantly, the task model is not a static representation and may be continually updated during reading (Winne and Hadwin, 2008; Britt et al., 2018). As readers obtain new information, their awareness of task demands may be refined, and the task model updated. This may include adjustments to goals, plans and strategies.

### The role of paraphrasing, bridging, and elaboration

Understanding how these processes may support task awareness requires an understanding of how they support text comprehension. Paraphrasing is the process of reframing content from a text in one’s own words and can be thought of as externalizing a reader’s understanding (McNamara, 2009). In the contexts of thinking aloud or self- explaining, paraphrasing is evidenced by reproducing semantic content analogous to the content in the sentence that was just read (e.g., McNamara, 2004: McMaster et al., 2012). While paraphrasing supports the comprehension of explicitly conveyed content, it may also facilitate retrieval of relevant information from the reader’s mental representation of prior discourse or relevant background knowledge (McNamara, 2004; McNamara et al., 2007). Bridging refers to a process of establishing how discourse segments are semantically connected (Singer, 1994; McNamara, 2004). While bridging can occur at the word level and in the context of resolving anaphora, texts often require establishing deeper semantic relationships, such causal (e.g., Singer and Halldorson, 1996) or logical (Lea, 1995) relationships. When thinking aloud or self-explaining, this often involves describing how content in the current sentence is related to prior discourse content (Magliano et al., 2011). Elaboration is a process of activating knowledge not explicitly conveyed in the text and applying that knowledge to the construction of meaning (McNamara, 2004; McNamara and Magliano, 2009b). When thinking aloud, elaborations are often a basis for explaining the texts (e.g., why events are happening, why information is being conveyed; Magliano et al., 1999). The extent that people engage in bridging, elaboration, and paraphrasing can vary among individuals and has been shown to be correlated with performance on literacy tasks (Magliano and Millis, 2003; Magliano et al., 2011; Clinton, 2015; Higgs et al., 2017).

These same comprehension processes are likely to support the construction of the task model that supports task awareness during reading. To build a task model, one needs to accurately represent propositions that reflect the content of the task as it was originally given (Britt et al., 2018), and therefore paraphrasing is likely a critical prerequisite to do so. These propositions may also serve as retrieval cues for readers’ knowledge related to how to complete tasks or relevant topic knowledge (Kintsch, 1998). Further, in the context of a task, readers engage in relevance processing in which readers identify content aligned with the task (which may or may not align with relevance to the main points intended by the author; McCrudden and Schraw, 2007) and direct more resources to processing the task relevant content. In this context, paraphrasing sentences aligned with the task should serve as a retrieval cue for the task model, and thereby increasing readers’ awareness of the task. The application of the task model to a text requires establishing semantic relationships between the task model and the texts, and therefore bridging could also increase the accessibility of the task model and awareness of the task. Finally, tasks that require problem solving inherently involve elaborative process (Britt et al., 2018; OECD, 2018), therefore, the extent that readers engage in elaborations should also affect what is represented in the task model and accessible to readers’ awareness.

### Overview of the present study

**FIGURE 1:**
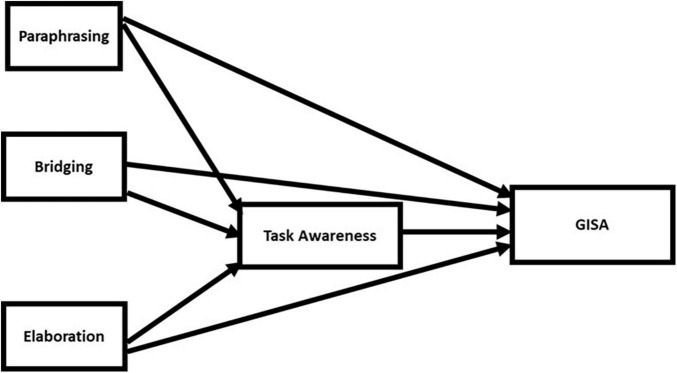
Task awareness mediation hypothesis.

The goal of this study was to assess the relationship between readers’ propensity to engage in these comprehension strategies, task awareness, and performance on an academic literacy task. Based on the discussion above, we propose and test a *task awareness mediation hypothesis* (see Figure 1). This hypothesis assumes that the comprehension strategies of paraphrasing, bridging, and elaboration support comprehension, and therefore the propensity to engage in these processes during reading (as evidenced in a think aloud task), is directly and positively correlated with comprehension outcomes (e.g., Kopatich et al., 2019; Magliano et al., 2020). This hypothesis also assumes that task awareness (as evidenced by one’s ability to describe why a particular text was given to help complete the task) is directly, positively correlated with comprehension outcomes. Finally, this hypothesis assumes that there are indirect paths from the comprehension strategies to comprehension outcomes through task awareness. This is based on the additional assumptions that the comprehension strategies affect the construction, activation and updating of the task model during reading, affecting task awareness which, in turn, determines how successfully those same processing strategies are utilized when comprehending a text to accomplish the task.

The task awareness mediation hypothesis was tested in the context of college readers who were administered assessments of (1) the propensity to engage in paraphrasing, bridging, and elaboration during reading (Magliano et al., 2011) and (2) a complex literacy task involving reading multiple documents to solve a problem (Sabatini et al., 2014). Participants were administered a scenario-based assessment (SBA), which is a standardized test of students’ ability to engage in a complex literacy task that requires problem solving (O’Reilly and Sabatini, 2013; Sabatini et al., 2020). For example, the two SBAs used in the present study tasked readers with either reading texts to decide if a wiki page needed to be corrected and how it should be updated or reading texts to understand a complex topic in preparation for an exam. The SBAs had characters that gave the tasks (i.e., instructor character) and student characters that helped the student keep the task in mind as they progress through the items. About halfway through each SBA, one of the characters asked why the group was given a specific text to read, and participants were asked to type their answers in a text box. The answers were scored to determine the extent that the task was mentioned and the level at which it was specified. This was the measure of task awareness used to assess the relationships between task awareness, performance on the SBA and readers’ propensity to engage in the three comprehension processes. Each SBA began with an assessment of prior knowledge of topics covered in the SBA. It is well established that prior knowledge of text topics supports comprehension (see McCarthy and McNamara, 2021 for an extensive review), and it is also positively correlated with performance on the SBAs (McCarthy et al., 2018). As described below, we conducted exploratory analyses to assess if prior knowledge influenced the relationships specified by the task mediation hypothesis.

The propensity to engage in paraphrasing, bridging, and elaboration was assessed with the Reading Strategy Assessment Tool (RSAT: Magliano et al., 2011). In the RSAT tool, participants read texts presented one sentence at a time on a computer and are periodically asked to respond to think aloud prompts. Computational algorithms are used to assess the think aloud protocols for evidence of paraphrasing, bridging, and elaboration and aggregated scores are used to provide a measure of the extent that students paraphrased, bridged, and elaborated when reading. RSAT scores are correlated with performance on standardized comprehension tests and experimenter generated tests of comprehension for texts not used in RSAT (Magliano et al., 2011). Importantly, these scores are also predictive of performance on the SBA, and in particular, it has been shown that elaboration is relatively more robustly correlated with performance on the SBA than bridging (Feller et al., 2020; Magliano et al., 2020). The present study also provided an opportunity to see if these relationships are replicated.

This study involved students enrolled in a reading and study strategies course. The SBA and RSAT were administered at the beginning and end of the course. This afforded the assessment of whether the results replicated across the two timepoints. In order to test the task awareness mediation hypothesis, we addressed four research questions and an exploratory question regarding the influence of prior knowledge:

RQ1: Is task awareness related to performance on a complex literacy task?
RQ2: Are reading comprehension strategies related to task awareness?
RQ3: What are the relative contributions of the comprehension strategies on complex literacy task performance?
RQ4: Is there evidence for the task mediation hypothesis and does it replicate across the two time points?

Given that prior work has shown a strong association between prior knowledge of the text content and the key variables in this study (Cromley and Azevedo, 2007; van den Broek, 2010; McCarthy et al., 2018) and that prior knowledge may facilitate constructing a task model (Britt et al., 2018), we added RQ5 to explore if the proposed hypotheses are supported after controlling for prior knowledge of the text content.

RQ5: Do prior knowledge scores account for any of the relationships specified in the task mediation hypothesis?

## Materials and methods

### Participants

A total of 359 undergraduate students from a large university in the Midwest United States provided data for this study. All participants were students enrolled in a “College Reading and Study Strategies” course. The majority of participants were first year students and included 252 participants who were enrolled in a developmental education program. The study consisted of two “in-class” sessions during the semester [one in the third week of the semester (*n* = 359) and one eight weeks later (*n* = 266)]. Students received class participation points for participating in the sessions but had the option to decline participation and complete alternate activities for class points. See Table 1 for demographics.

**TABLE 1:** Demographic information for participants.

Participant information	Total	Proportion
**Developmental enrollment**		
DE	252	0.70
Not DE	62	0.17
No info	45	0.13
**Sex**		
Female	79	0.40
Male	115	0.59
Other	1	0.01
No response	1	0.01
**First language**		
English	174	0.89
Not English	19	0.10
No response	3	0.02
**Race/Ethnicity**		
Black/African American	102	0.52
White	50	0.26
Asian	6	0.03
Hispanic/Latino	35	0.18
American Indian/Alaska native	0	0.00
Native Hawaiian/Pacific Islander	1	0.01
Other	7	0.04
**Age range**		
18–20	174	0.89
21–30	9	0.05
No response	13	0.07

Only 196 completed the demographic survey administered at the end of T2. Developmental enrollment information was available for the majority of participants.

### Transparency and openness statement

The original sample size for the data collection associated with this study was intended to be 500 participants. This was based on a power analysis for a study designed to test the efficacy of the course. However, there were practical constraints (i.e., changes to the course structure) that led to the termination of data collection before that sample size was reached. The study reported in this paper was conceptualized after data collection was completed. The measures described below were chosen to support the original goal to test the efficacy of the course but were determined to be relevant to addressing the research questions of the present study. In addition to the measures reported in this study, participants were administered the Study Aid and Reading Assessment (Sabatini et al., 2015), items adapted from the Reading Motivation Measure (Kingston et al., 2017), the Situated Reading Motivation Measure adapted from the Experience Sampling Method (Csikszentmihalyi and Larson, 2014), the Metacognitive Awareness Inventory (Schraw and Dennison, 1994), and the Metacognitive Awareness of Reading Strategies Inventory (Mokhtari and Reichard, 2002).

### Measures

The measures of academic reading and comprehension strategies used in the current study were obtained at two time points. Time 1 (T1) occurred in the third week of the semester and Time 2 (T2) occurred 8 weeks later. Measures of task awareness were grounded in the academic reading assessment.

#### RSAT

Comprehension strategies were assessed at T1 and T2 with the Reading Strategy Assessment Tool (RSAT; Magliano et al., 2011). RSAT is a computer-based assessment tool that provides measures of processes supporting comprehension of texts, in particular (1) paraphrasing, (2) bridging, and (3) elaborative inferences.

The RSAT measures were obtained using a variation of think-aloud instructions in which participants produce typed, open-ended verbal protocols. Texts were presented one sentence at a time and participants advanced to the next sentence at their own pace. Only the current sentence was visible to participants. After target sentences, participants saw the prompt “What are you thinking now?” appear on the screen and typed responses into a text box beneath the prompt.

Reading Strategy Assessment Tool scored the protocol using computational algorithms, based on key word matching, to assess the extent to which words from a participant’s protocol overlapped with words from the text (see Magliano et al., 2011). The paraphrasing score was based on overlap between words in the student’s protocol and words that appeared in the sentence prior to the prompt (i.e., the sentence that was just read). The bridging score was generated based on the number of content words from prior sentences (i.e., sentences prior to the sentence that was read just before the prompt). The elaboration score was generated based on the number of content words in the participant’s response that were not present in the prior discourse context.

Despite the simplicity of the scoring algorithms, RSAT has shown good construct validity with moderate to high correlations between computer scores and human judgments of the presence of paraphrasing (*r* = 0.75), bridging (*r* = 0.71), and elaboration (*r* = 0.50) (Magliano et al., 2011). Test-retest reliability of the automated scores were high, particularly when the open-ended nature of the assessment is considered (*r*’s ranging from 0.59 to 0.79). Additionally, like human judgments of think-aloud protocols (Magliano and Millis, 2003), RSAT scores have been shown to be predictive of performance on standardized tests and experimenter generated tests of comprehension (Magliano et al., 2011).

In the current study, participants read a set of two texts in RSAT at each time period. Each of the two text sets consisted of a science and a history text. The presentation of the text sets was counterbalanced across T1 and T2 and within each set, the two texts were presented in a randomized order. In text set 1, participants read a history text (“Louis XVI and the French Revolution,” 19 sentences) and produced verbal protocols at 6 locations, and a science text (“The Power of Erosion,” 22 sentences) in which they produced protocols at 7 locations. Text set 2 consisted of a history text (“Spanish Civil War,” 21 sentences) in which students produced verbal protocols at 7 locations, and a science text (“Cancer,” 27 sentences) in which they produced protocols at six locations.

Prior to reading the texts, participants were given instructions and then engaged in a practice text to familiarize themselves with the presentation format and responding to the prompt. Participants were instructed that when they saw the prompt “What are you thinking now?,” they were to type their thoughts about their understanding of what they had just read in terms of what they had already read and what they know about the topic. During the practice, participants were given feedback when their responses were less than five words (i.e., “We are interested in your thoughts about the texts, in your responses to the prompts, please tell us more about your understanding of what you are reading”). After the practice, participants read the two experimental texts. No feedback was provided during the experimental texts.

#### GISA

The Global, Integrated, Scenario-based Assessment (GISA; Sabatini et al., 2019) is a scenario-based assessment (Sabatini et al., 2020) that was originally developed for high school students but adapted for this study for use with early-stage college students. The GISA provided an assessment of academic reading as well as a measure of prior knowledge and task awareness.

Two forms of the GISA were used in the current study. Participants received different forms at T1 and T2 and the form initially completed at T1 was counterbalanced across participants. One version involved a scenario in which students were asked to update and correct a wiki about the Mona Lisa. Through interaction with various texts and the GISA agents, students completed tasks that included identifying the problem with the wiki (i.e., it only presented one of many theories about the identity of the person depicted in the Mona Lisa) and suggesting how to update the wiki. The second form involved a scenario in which students form a study group to prepare for an exam covering “problems associated with invasive species and potential solutions for dealing with them.” As with other GISA forms, students interacted with the student and teacher GISA agents and various texts to perform tasks to understand the problems and solutions associated with invasive species.

##### GISA academic literacy task

Academic reading was assessed at T1 and T2 using the GISA. In the GISA, items are grounded in an academically authentic task (e.g., the need to correct a wiki on a historical topic). GISA scenarios involve simulated teacher and student agents that contextualize each item in the task, help to structure and scaffold the tasks, as well as provide test takers an opportunity to identify and correct errors expressed by the simulated students. Unlike many off-the-shelf reading assessments that measure the piece-meal understanding of single texts, GISA provides test takers with a realistic, domain-specific purpose for reading a collection of sources and materials. This allows for the measurement of skills associated with higher-level comprehension such as knowledge of text structure, evaluation, application, perspective taking and integration of information in service of completing a goal through GISA (see O’Reilly and Sheehan, 2009; Bennett, 2011; O’Reilly and Sabatini, 2013; Sabatini et al., 2013, 2018).

The GISA has been shown to be reliable in elementary through high school populations as evidenced by good internal consistency (Cronbach’s α > 0.80; O’Reilly et al., 2014) and test-retest reliability (*r* = 0.87; Sabatini et al., 2014). Additionally, the GISA has robust correlations with other reading measures such as English language arts state test scores ranging from 0.52 to 0.68 (O’Reilly et al., 2014) and correlates with measures of deep understanding including academic vocabulary, complex reasoning, and perspective taking (LaRusso et al., 2016). The items cover a broad range of difficulty with no apparent floor or ceiling effects when used with intended populations (see O’Reilly et al., 2014; Sabatini et al., 2014; McCarthy et al., 2018).

The GISA score for each form was a single scaled score based on 24 selected response items. These comprehension items included items assessing comprehension of the individual texts, as well as items that required the reader to apply textual information beyond the text, and to integrate across texts. These items are part of a larger collection of scenario-based reading comprehension items. The items in the larger pool were calibrated using a multigroup extension of the item response theory (IRT), two-parameter logistic model. This was done to place all items and scores on a common scale. Scale scores are computed by transforming expected *a posteriori* ability estimates—based on the IRT item parameters—to a metric with a population mean of 1,000 and a standard deviation of 500. Due to the time limitation of a single class period, some students did not complete the GISA assessment. However, many were close to finishing. For the students who had completed enough of the assessment, an adjusted GISA score was calculated using the method described above.

##### GISA task awareness measure

Both GISA forms included an open-ended question that provided a basis for assessing task awareness. The open-ended question was posed by a student character asking why the students have been given a specific text (i.e., “Why do you think Dr. Henson gave us this text to read?;” “Why do you think Andrea wanted us to read this excerpt?”). The prompt appeared immediately after the student had an opportunity to read the text and the text was available to the student while they responded to the question prompt. Responses to the open-ended question in the GISA were coded for evidence of task awareness. To answer the question, participants needed to be able to express how the text related to the overall task (i.e., correcting the wiki). In the Invasive Species form, the text was relevant to the goal of understanding problems and solutions related to invasive species. The text was an excerpt of a report providing a specific example of an invasive species invading a lake and the implementation of a solution. The texts in which the task awareness question prompt appeared gave significant new task-relevant information providing an opportunity to assess the readers’ task awareness. In the Davinci form, the prompt text came immediately after students had read the wiki that they were tasked with correcting. At this point, they had only been told that there were inaccuracies in the Mona Lisa page of the wiki but did not know the nature of the inaccuracies. The text provided an account of the identity of Mona Lisa that contradicted the account in the wiki. For the Invasive Species form, the prompt text came later in the scenario. The task was to understand problems and solutions related to invasive species. Prior texts had given detailed information about the problems related to invasive species but had only addressed solutions in the form of general goals (“manage species and ecosystems” and “restore species and ecosystems”). The prompt text provided the reader with a specific example of solutions in the form of a report detailing the implementation and results of an invasive species management program.

Participants’ responses were coded on the extent to which they reflected awareness of the task. Task awareness was scored on three levels (2 = articulates specific information about the task; 1 = articulates general information about the task; 0 = did not articulate any information about the task). Responses were given a score of 2 on task awareness for a direct mention of the specific task or an indirect statement revealing how the text related to the task. For example, in the Davinci form they would receive a 2 if they mentioned the specific task of correcting the wiki (e.g., “To fix the wrong information that is in there”) or if they indicated that there was a controversy that required resolution (e.g., “Because it’s telling us something different now,” “It provides another theory about the identity of the Mona Lisa”). For the Invasive Species form, a 2 would be given for mentioning the goal of understanding problems or solutions related to invasive species (e.g., “So we can learn how invasive species can have an impact on native species,” “It shows how they got rid of a invasive species”). Responses were given a score of 1 on task awareness if they indicated a more general task such as information gathering without explaining the purpose of gathering information (e.g., “So we can learn more additional information”). Responses were given a score of 0 on task awareness if they did not mention any task information (e.g., “To confuse us”) or is uninformative (e.g., “So that we can read the passage”). For the two forms, interrater reliability on the two dimensions was acceptable (DaVinci: κ = 0.83 for task awareness; Invasive Species: κ = 0.83 for task awareness).

##### GISA prior knowledge measure

The measure of participants’ prior topic knowledge for each of the two topics was embedded in the GISA SBAs and integrated as part the scenario. The prior knowledge items were completed at the beginning of the scenario prior to reading texts or completing any other items in the assessment. The items included a topical vocabulary test and multiple choice items assessing factual knowledge of the topic. For the vocabulary-based assessments, students saw a list of words and indicated if the word was (1) related to the topic (2) not related to the topic or (3) I don’t know. Responses of “I don’t know” were counted as incorrect. Each form had 44 vocabulary items and 13 multiple choice items.

### Procedure

The study consisted of two sessions that occurred approximately 8 weeks apart. All measures were computer-based and accessed via web links. Instructions for each measure were provided on the websites. Participants completed both sessions in a group setting at a computer lab during class time. Sessions began with the RSAT followed by the GISA and the majority of students completed the assessments within the 75-min class period.

### Analysis

We conducted analyses using the psych package in R (Revelle, 2019). Initial regression analyses were conducted to confirm the relationships assumed in the task awareness mediation hypothesis. We first examined the associations between reading strategies and academic reading performance at each time point. The associations were tested with each strategy as a predictor in separate models as well as in a model with all three strategies tested as simultaneous predictors of comprehension. Conducting analyses in separate and simultaneous models provided the opportunity to assess how the Beta weights change across analyses, and thus how each strategy might uniquely contribute to outcomes. We then estimated indirect effects of the strategies to comprehension through task awareness for each of the separate models and the simultaneous model. Indirect effects between the comprehension strategies and performance on GISA provide evidence in favor of the task awareness mediation hypothesis and against the independence hypothesis.

## Results

### Research question 1 (RQ1)

Research question one pertained to confirming the expected relationships between task awareness and performance on a complex literacy task (RQ1). Descriptive statistics for the study measures can be found in Table 2 and correlations between study measures can be found in Table 3. As can be seen in the correlation table (Table 3), Task Awareness was positively related to literacy task performance in GISA at both time 1 (T1) and time 2 (T2) and the magnitude of the correlation coefficients were comparable. The bivariate correlations support the expected relationships between task awareness and reading performance on the GISA.

**TABLE 2:** Descriptive statistics for the measures.

	T1 mean	T1 std. dev.	T2 mean	T2 std. dev
GISA score	1,021.22	73.27	997.92	67.56
Task awareness	1.08	0.72	0.98	0.65
Paraphrase	1.17	0.57	1.12	0.59
Elaboration	2.83	1.5	2.24	1.31
Bridging	1.56	0.91	1.33	0.85
GISA prior knowledge	24.24	7.68	25.4	7.89

**TABLE 3:** Correlations at Time 1 and Time 2.

Time 1 correlations						
s	GISA score	Task awareness	Paraphrase	Bridge	Elaboration	GISA prior knowledge
GISA score	–					
Task awareness	0.39**	–				
Paraphrase	0.22**	0.11	–			
Bridge	0.15**	0.03	0.67**	–		
Elaboration	0.18**	0.14*	−0.01	0.25**	–	
GISA prior knowledge	0.06	0.10	0.05	0.12*	0.14**	–
**Time 2 correlations**						
	**GISA score**	**Task awareness**	**Paraphrase**	**Bridge**	**Elaboration**	**GISA prior knowledge**
GISA score	–					
Task awareness	0.37**	–				
Paraphrase	0.20**	0.28**	–			
Bridge	0.15*	0.23**	0.72**	–		
Elaboration	0.31**	0.22**	0.11	0.23**	–	
GISA prior knowledge	0.22**	0.156*	0.10	0.10	0.13*	–

*p < 0.05, **p < 0.01.

### Research question 2 (RQ2)

Research question two pertained to confirming the expected relationships between task awareness and reading comprehension strategies (RQ2). The bivariate correlations (see Table 3) tentatively support the expected relationships between comprehension processes and task awareness. Elaboration was significantly correlated with task awareness at both T1 and T2. For paraphrasing, the correlation with task awareness was a non-significant trend at T1 (*p* = 0.053) but was significant at T2. The correlation between task awareness and bridging was not significant at T1 but was significant at T2. Importantly, the support for the expected relationships outlined in research questions 1 and 2 suggest that testing the task mediation analyses is warranted.

### Research question 3 (RQ3)

The next set of analyses focused on assessing the relative contributions of the three comprehension strategies on performance on the GISA (RQ3). To confirm the assumption that readers’ propensity to engage in these comprehension strategies would predict literacy task performance, regression analyses were conducted for each time point. Based on prior work, it was expected that all three strategies would positively predict GISA performance at both T1 and T2. In order to evaluate the relationships between strategies and GISA across the models each strategy was tested in a separate model and then together in a simultaneous model (see Table 4 for estimates).

**TABLE 4:** Regression estimates for separate and simultaneous models of comprehension processes predicting SBA literacy task performance at two time points.

	Time 1				Time 2			
	T1 estimate	T1 SE	T1 *p*-value	T1 model R2	T2 estimate	T2 SE	T2 *p*-value	T2 model R2
Separate models								
Paraphrasing	28.38	7.14	<0.001	0.05	22.55	7.04	<0.01	0.04
Bridging	12.22	4.52	<0.01	0.02	12.17	4.98	0.02	0.02
Elaboration	8.96	2.83	<0.01	0.03	16.31	3.2	<0.001	0.09
Simultaneous model				0.08				0.12
Paraphrasing	35.43	9.71	<0.001	–	23.6	9.68	0.02	–
Bridging	−6.9	6.31	0.28	–	−5.1	6.93	0.46	–
Elaboration	9.99	2.96	<0.001	–	15.74	4.84	<0.001	–

Separate models are three regressions with a single predictor and simultaneous model includes all three predictors at once.

When tested as separate equations, paraphrasing, bridging, and elaboration positively predicted reading comprehension at both time periods. The R^2^ for the separate paraphrasing and bridging models were stable across T1 and T2 (T1 R^2^: paraphrasing = 0.05, bridging = 0.02; T2 R^2^: paraphrasing = 0.04, bridging = 0.02) but increased for elaboration between T1 and T2 (T1 *R*^2^ = 0.03; T2 *R*^2^ = 0.09).

Next, we tested a multiple regression model with all strategies entered as simultaneous predictors in order to account for common variance and understand the unique relationships each strategy had with GISA. In this simultaneous model, paraphrasing and elaboration were significant predictors at T1 and T2, but bridging was not. The R^2^ for the simultaneous models, were stable across T1 and T2 (T1: *R*^2^ = 0.08, T2: *R*^2^ = 0.12).

That bridging was no longer a significant predictor of GISA performance in the simultaneous model, replicated Magliano et al. (2020), which showed that elaboration was a more robust predictor of GISA performance than bridging in the context of a simultaneous model. This suggests that the part of bridging that contributes to academic literacy performance is shared with the other reading strategies. It also suggests that paraphrasing and elaboration each have unique variance that contributes to reading comprehension beyond the contributions of other reading strategies in the model.

### Research question 4 (RQ4)

To examine the task mediation hypothesis (RQ4), indirect effects analyses were conducted for each comprehension strategy in a separate model and with the three predictors combined in a simultaneous model. Analyses were conducted using the psych package mediation function in R (Revelle, 2019). Estimates for the separate models and simultaneous models are shown in Table 5.

**TABLE 5:** Estimates from T1 and T2 separate indirect effects models for each reading strategy predicting SBA literacy performance.

	Time 1				Time 2			
	Estimate	SE	*P*-value or CI	Model R2	Estimate	SE	*P*-value or CI	Model R2
Separate models								
Paraphrasing (C’)	22.86	6.2	<0.001	0.17	11.21	5.8	0.054	0.15
Task awareness (b)	36.05	4.96	<0.001		36.11	5.3	<0.001	
Paraphrase (a)	0.14	0.07	0.037		0.32	0.06	<0.001	
Ab	4.97	(sd = 2.7)	[−0.08, 10.58]		11.43	(sd = 3.47)	[5.39, 18.77]	
Bridging (c’)	11.48	3.92	0.004	0.16	5.49	4.01	0.172	0.15
Task awareness (b)	37.88	4.97	<0.001		37.4	5.23	<0.001	
Bridging (a)	0.02	0.04	0.619		0.18	0.04	<0.001	
Ab	0.79	(sd = 1.73)	[−2.64, 4.34]		6.72	(sd = 2.58)	[2.37, 12.40]	
Elaboration (c’)	5.94	2.4	0.014	0.15	11.99	2.52	<0.001	0.19
Task awareness (b)	36.56	5.02	<0.001		33.97	5.06	<0.001	
Elaboration (a)	0.06	5.02	0.01		0.11	0.03	<0.001	
Ab	2.37	(sd = 1.16)	[0.15, 4.72]		3.59	(sd = 1.64)	[0.8, 7.27]	

c’ = direct effect of comprehension strategy on outcome; a = effect of comprehension strategy on mediator; b = effect of mediator on outcome; ab = indirect of comprehension strategy on outcome effect through mediator.

First, we will consider the separate indirect effects models at T1 and T2. In all models, task awareness significantly predicted GISA performance. In the bridging model at T1, only the direct effect of bridging on GISA performance was significant. Bridging did not significantly predict task awareness and the indirect effect of bridging through task awareness was not significant. The pattern at T2 differed. The direct effect of bridging on GISA performance was not significant this time. However, bridging significantly predicted task awareness and the indirect effect of bridging through task awareness on GISA was significant. In the separate elaboration model at T1 all effects were significant. Elaboration directly predicted GISA performance and task awareness and the indirect effect of elaboration through task awareness to GISA performance was significant. The same pattern of results was found at T2. In the separate paraphrasing model at T1, paraphrasing directly predicted task awareness and GISA performance. However, the indirect effect of paraphrasing through task awareness on GISA was not significant. At T2 paraphrasing directly predicted task awareness, but the direct relationship with GISA performance was not significant (*p* = 0.054). However, this time there was a significant indirect effect of paraphrasing through task awareness. The R^2^ for the separate T1 and T2 mediated models suggested that more variance was explained in GISA performance than in the separate models that did not include task awareness (T1: paraphrasing *R*^2^ = 0.17, bridging *R*^2^ = 0.16, elaboration *R*^2^ = 0.15; T2: paraphrasing *R*^2^ = 0.15, bridging *R*^2^ = 0.15, elaboration *R*^2^ = 0.19). The separate models provided support for the task mediation hypothesis for Elaboration at both time points. However, the separate models provided inconsistent support for the task mediation hypothesis for bridging (T2 only) and paraphrasing (T2 only).

**FIGURE 2:**
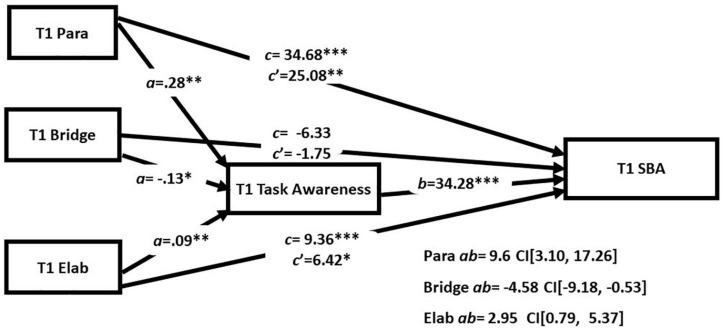
Simultaneous mediation analyses at T1. *p < 0.05, ^**^p < 0.01, and ^***^p < 0.001.

**FIGURE 3:**
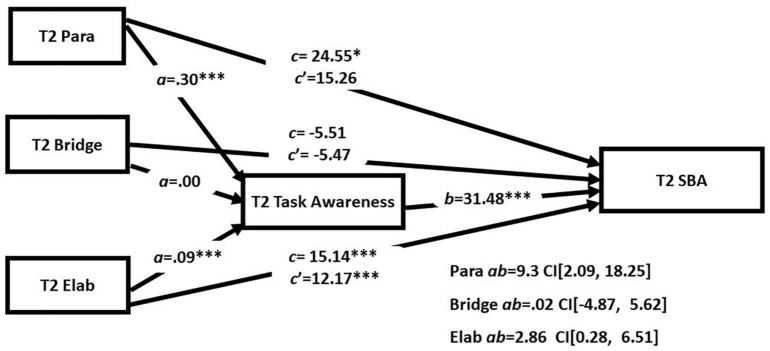
Simultaneous mediation analyses at T2. *p < 0.05 and ^***^p < 0.001.

Next, we examined the task mediation hypothesis in indirect effects models that included the three strategies simultaneously in order to assess the extent to which the reading strategies had unique relationships with academic reading outcome and task awareness (see Table 5 and Figure 2 for T1 and Figure 3 for T2). There were direct effects of both paraphrase and elaboration scores at T1 and T2 on performance on GISA. Importantly both models also showed evidence of indirect effects of paraphrase and elaboration scores involving task awareness. The primary difference across the models was that bridging was significantly related to task awareness at T1 but not T2. The results support the task mediation hypothesis and suggest a strong case for task awareness as an indirect route for the effect of paraphrasing and elaboration on comprehension.

### Research question 5 (RQ5)

In our final analyses, we assessed whether prior knowledge scores accounted for any of the relationships specified in the task mediation hypothesis. Prior knowledge related to the reading topic did not differ significantly across the two assessment periods, *t*(257) = −1.94, *p* = 0.053; (T1: *M* = 24.39, SD = 7.54, T2: *M* = 25.47, SD = 7.87). Interestingly, prior knowledge was not correlated with GISA performance scores at the first assessment period (*r* = 0.06, *p* = 0.30) and only showed a small but significant association with GISA performance at the second assessment period (*r* = 0.22, *p* ≤ 0.001).

**FIGURE 4:**
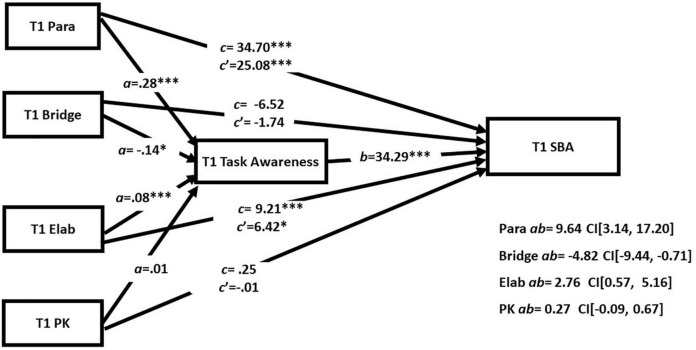
Simultaneous mediation analyses with prior knowledge at T1. *p < 0.05 and ^***^p < 0.001.

**FIGURE 5:**
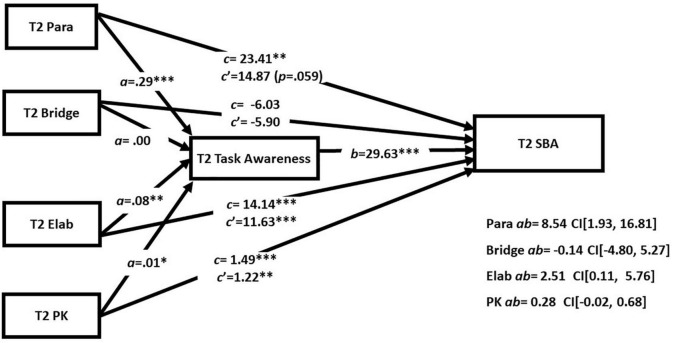
Simultaneous mediation analyses with prior knowledge at T2. *p < 0.05, ^**^p < 0.01, and ^***^p < 0.001.

Analyses for the primary hypotheses (RQ4) were re-analyzed while controlling for prior knowledge (see Figure 4 for T1 results and Figure 5 for T2 results). As expected from the small correlations with GISA performance, the same pattern of results was found when topic prior knowledge was accounted for. At T1 there were no significant effects of prior knowledge on GISA performance or task awareness. At T2 there was a significant positive effect of prior knowledge on task awareness (*a* = 0.01, *p* = 0.02) and of prior knowledge on GISA performance (*c’* = 1.22, *p* = 0.003). However, the indirect effect of prior knowledge on GISA performance was not significant.

## Discussion

While it is well established that reading tasks affect literacy outcomes (e.g., Britt et al., 2018), how they do so is less understood. Theories of task-oriented reading (Rouet, 2006; McCrudden and Schraw, 2007; Rouet and Britt, 2011; Britt et al., 2018) and self-regulated learning (e.g., Winne and Hadwin, 1998, 2008) explicitly or implicitly assume that awareness of the task affects performance when engaged in problem solving with texts.

The present study was conducted to develop a better understanding of the relationships between task awareness, comprehension and cognitive processes that support constructing mental representations. Specifically, we postulated that task awareness contributes to performance on a complex literacy task, and that it is supported in part by the same processes that are involved in constructing a coherent mental model for a text. In the current study we looked at paraphrasing, bridging and elaboration processes. Further, we proposed and tested the task awareness mediation hypothesis that task awareness mediates the relationship between these processes and comprehension outcomes on a complex literacy task (GISA SBA) and tested its components across four research questions and one exploratory question.

In RQ1 and RQ2, we verified assumptions of the task awareness mediation hypothesis. For RQ1 we found that task awareness was positively correlated with performance on the GISA SBA at both time points, and to a similar magnitude. To our knowledge, this is one of the few studies to show that task awareness is related to a comprehension outcome. Much of the research on task awareness has investigated it in the context of its role in helping students to focus attention and regulate application of strategic processing on task relevant content (Schellings et al., 1996; Winne and Hadwin, 1998, 2008; Schellings and Broekkamp, 2011). Some of these studies used think-aloud methods during a task in which readers selected main points based on different perspectives such as importance to the author, to an imaginary biology teacher or of personal interest (e.g., Schellings et al., 1996; Schellings and Broekkamp, 2011). While these measures of task awareness were not tied directly to comprehension measures, Schellings et al. (1996) found performance on the selection task was positively related to final course grades. Other work has looked at the related constructs, task understanding and task interpretation (e.g., Hadwin et al., 2009; Lawanto et al., 2013; Rivera-Reyes et al., 2016). Much of this work has used task questionnaires that probed implicit, explicit, and socio-cultural aspects of task models in the context of a class project (e.g., a complex academic activity extended over time). Some of the questions probing implicit aspects of the task model included questions related to understanding task purpose, which may be the most clearly aligned with our measure of task awareness. However, the implicit questions also probe other aspects of task understanding such as resources needed and relevant course concepts important to task completion. These studies have found that task understanding, and in particular the implicit aspects, predicted both task and course performance (e.g., Miller, 2009; Oshige, 2009; Rivera-Reyes et al., 2016) and a post-task measure of conceptual understanding (Rivera-Reyes et al., 2016).

With respect to RQ2, we found the bivariate correlations partially supported relationships between task awareness and comprehension processes. Task awareness was consistently correlated with elaboration at both time points. For paraphrasing, the correlation with task awareness at T1 did not reach significance (*p* = 0.053) but was significant at T2. For bridging, the correlation with task awareness was only significant at T2. It is well established that elaboration of discourse segments affects their accessibility during reading (e.g., Albrecht and Myers, 1998). We suspect that task awareness similarly increases to the extent that the task model is elaborated because of an increased accessibility of propositions represented in a task model. Paraphrasing may be important for building a task model, by helping readers to accurately represent propositions in task instructions. Further, these propositions may serve as retrieval cues for knowledge related to how to complete the task or relevant topic knowledge. It is also possible that paraphrasing task relevant sentences during reading may serve as a retrieval cue for the task model, and thereby increases readers’ awareness of the task as they read. Bridging was only positively correlated with task awareness at T2. While bridging is important for supporting comprehension (e.g., see Graesser et al., 1994 for an extensive review), we have found that it is relatively less correlated with performance on complex literacy tasks as assessed in GISA (e.g., Feller et al., 2020; Magliano et al., 2020).

For RQ3, we examined the relative contributions of the three comprehension strategies on academic literacy task (GISA) performance. When the comprehension strategies were tested in separate models, all three comprehension strategies significantly predicted GISA performance at both time points. However, when entered simultaneously in the same model, paraphrasing and elaboration were significant predictors at T1 and T2, but bridging was not a significant predictor at either time point. The results replicated those of prior studies (Feller et al., 2020; Magliano et al., 2020) showing that elaboration is more strongly related to performance on a complex comprehension task than bridging, and in particular performance on GISA. The present study found consistent evidence of this at both T1 and T2. Together, these findings may stem from the nature of the items on GISA, which require one to demonstrate that they can accurately understand text content and successfully engage in reasoning with and beyond the explicitly conveyed content in the texts (O’Reilly and Sabatini, 2013; Sabatini et al., 2013). While these results replicate those of prior studies and provide a within study replication, we hesitate to overgeneralize that bridging is not supportive of a complex reading task. If such a task required activities that involved establishing semantic relationships between texts, for example, we suspect bridging would support such activities.

Addressing RQ4 was the primary purpose of this study and we found evidence to support the task awareness mediation hypotheses across both time points. We found direct and indirect paths between elaboration and performance on GISA at both time points. For paraphrasing, we found both direct and indirect paths to GISA at T1. At T2 the indirect path to GISA was significant, but the direct path did not reach significance (*p* = 0.056). As such, task awareness partially explains why the propensity to paraphrase and elaborate supports complex literacy task performance. We did find evidence of an indirect path involving bridging at the first time point; however, the coefficient estimate was negative (*B* = −4.58). Given that all bivariate associations were positive, and the models involving bridging when tested separately also showed positive path coefficients, the negative values observed in the simultaneous model are likely an artifact from a suppression effect. Importantly, this study demonstrates a within study replication of the test of the task mediation hypothesis that is remarkably consistent across the two timepoints. This is an important aspect of this study given the well documented challenges of replication in the psychological sciences (Maxwell et al., 2015).

How might task awareness explain relationships between the propensity to paraphrase and elaborate and performance on GISA? In addition to contributing to the initial construction of a task model, these comprehension processes may also affect the updating of the task model during reading. In general, comprehension strategies are dynamically employed over reading (McNamara and Magliano, 2009a). That is, readers differentially use paraphrasing, bridging, and elaboration as they progress through a text and construct a mental model of the text. These strategies are deployed based on semantic features that are changing across a text (e.g., changes in causal relationships reflected in the text content, changes in argument overlap, the introduction of new concepts) and metacognitive states that change over the course of reading (Magliano et al., 1999; McNamara and Magliano, 2009a). A reader’s level of awareness of the task may also dynamically change as one progresses through the task, and the extent that readers paraphrase and elaborate may help to enhance task awareness. Paraphrasing may support task awareness by strengthening propositions in the task model that reflect memory for the task and how it might be related to content being read. Elaborative processes may support updating the task model based on content that is being read, which would require one to activate content from the task model enhancing its accessibility.

Readers’ mental representations of tasks play an important role in theories of task-oriented reading and theories of self-regulated learning (e.g., Britt et al., 2018). Given that the comprehension strategies investigated in the current study have been shown to play an important role in constructing a mental representation of text content during reading (e.g., Graesser et al., 1994), we proposed that they may also support a reader’s mental representation of the task affecting their task awareness during reading. The results of the current study suggest that readers’ propensity to engage in paraphrasing and elaborative processes may play an important role in constructing and updating a task model that supports task awareness during reading. Readers who tend to utilize these processes to a lesser extent may construct more impoverished task models compared to readers who tend to engage these processes more frequently. Task information that is not represented in a reader’s task model will not be accessible during reading (i.e., task awareness). Readers who do not have a clear task representation are less likely to be able to regulate their reading and effectively use strategic processes in service of the task. For example, they would be less likely to be able to identify task relevant text content and to process this content more deeply in a manner that would support task success (Britt et al., 2018). Indeed, this may influence how effectively readers can deploy paraphrasing, bridging and elaboration processes during reading to construct a mental representation of the text(s) that supports task success. As such, understanding factors that contribute to task awareness can help us to understand how to support readers as they face increasingly complex reading tasks.

Although it is agreed that task awareness plays an important role in comprehension and self-regulated learning (Winne et al., 2002; Butler and Cartier, 2004; Schellings and Broekkamp, 2011; Britt et al., 2018) there has been little research that has measured the construct and assessed its relationship to comprehension outcomes (e.g., Llorens and Cerdán, 2012; List et al., 2019). There is a great deal of difficulty in measuring such a complex construct that interacts dynamically with many processes to contribute to comprehension, and ultimately to task performance. Think aloud studies have shown evidence of differing degrees of task awareness through its influence on processes such as the selection of task relevant text (e.g., Schellings and Broekkamp, 2011). Other work has measured different aspects of a learners’ task understanding using task grounded questions given before, during, or after engaging in the task (e.g., Schraw, 1998; Miller, 2009; Oshige, 2009; Rivera-Reyes et al., 2016; Schoor et al., 2021; Kielstra et al., 2022).

In this study we measured task awareness by asking readers to explain why a specific text was relevant to their task. To answer, readers would need to access their mental representation of the task and assess how the text would contribute to accomplishing their purpose or the end goal state (the task outcome). It is remarkable that this simple measure of readers’ ability to explicitly produce information about the task purpose was predictive of comprehension performance and also showed structural relationships with general comprehension processes. However, measuring in classroom room settings, with complex academic activities that extend over time may require more complex instruments that capture other aspects of readers task representations (e.g., Miller, 2009; Oshige, 2009; Rivera-Reyes et al., 2016). Our measure is applicable in situations that involve reading to perform a task. However, it is essentially asking the reader to assess the relevance of a resource to their task. In other learning situations that do not involve reading to the same extent, questions probing the relevance of a resource/resources may also help to reveal task awareness.

### Limitations

One potential limitation of our approach is that we measured task awareness only once while participants completed a task. As readers’ task models are continually being updated, it would be ideal to obtain multiple measures of task awareness. However, in the context of this study, there was the possibility that prompting students multiple times would signal to participants that they should be paying attention to the task, and therefore compromise our ability to assess spontaneous task awareness. Developing approaches that enable one to assess task awareness at multiple timepoints are warranted but doing so surreptitiously will present challenges. On the other hand, it is possible that overtly asking students to reflect on the tasks as they progress through a text(s) could serve as a simple intervention. The results of the present study suggest that if doing so prompts students to be more aware of the task, then their performance on the task will benefit. Indeed, questions and checklists given prior to reading can encourage readers to elaborate their task models and increase task performance (e.g., Ayroles et al., 2021; Kielstra et al., 2022).

Another potential limitation is that we used measures of comprehension processes obtained from think-aloud protocols to predict performance on GISA, but the think aloud data was produced in a separate measure (RSAT). The RSAT provides measures of readers’ propensity to engage in comprehension processes, however, relationships between think aloud measures and outcome measures are typically more robust when they involve the same texts (e.g., Kopatich et al., 2019). Another limitation to consider is that students had to comprehend the text before they could provide a response about how it related to the task. Although the task awareness score was based on the extent to which the reader specified the relationship of the text to the task (requiring task awareness), other high level comprehension skills captured in the GISA academic literacy score could also have contributed to the reader’s ability to understand how the text supported the task. Although this possibility could not be accounted for in the current design, future research should consider ways to differentiate the contributions of task awareness from the contributions of literacy skills captured in the outcome measure. Finally, it is important to acknowledge that the variance explained by some of the relationships are relatively small.

## Conclusion

In conclusion, the results of this study suggest that the comprehension processes that support the construction of a task model and task awareness may be similar to those that support constructing mental models for texts (Britt et al., 2018). Constructing mental models likely relies on a common set of processes (Gernsbacher et al., 1990; Kintsch, 1998). Importantly, the results of the current study show that there are complex relationships between comprehension strategies, task awareness, and performance on an academic literacy task and illustrates the importance of understanding direct and mediational relationships, and that task awareness is an important factor to consider. Other researchers have argued that there are complex, structural relationships between proficiencies of the reader, language-specific skills, knowledge, inference processes, strategies, metacognitive monitoring, and comprehension performance (Cromley and Azevedo, 2007; Kopatich et al., 2019). This examination of the relationships between paraphrasing and bridging and elaborative inferences, task awareness and performance on a complex literacy task suggests further work is warranted to better understand these relationships and to identify additional factors that contribute to task awareness and comprehension performance across different reading contexts. These contexts should include different types of literacy tasks with different levels of complexity, different topic domains, and different student populations. Further, as suggested in the discussion of limitations, future work should continue to refine methods of measuring task awareness and explore the potential to leverage such measures as learning tools.

## Data availability statement

All data have been made publicly available at the Open Science Framework (OSF) repository and can be accessed at https://osf.io/wu237/.

## Ethics statement

The studies involving human participants were reviewed and approved by the Office of Research Compliance, Integrity and Safety (ORCIS)-Institutional Review Board Northern Illinois University. The patients/participants provided their written informed consent to participate in this study.

## Author contributions

KH, AS, RK, DF, and JM contributed to the design of the work and acquisition and interpretation of the data. CG, AS, KH, and RK contributed to the analysis. All authors contributed to the article and approved the submitted version.
